# Neuromagnetic Beta-Band Oscillations during Motor Imitation in Youth with Autism

**DOI:** 10.1155/2018/9035793

**Published:** 2018-07-25

**Authors:** I. Buard, E. Kronberg, S. Steinmetz, S. Hepburn, D. C. Rojas

**Affiliations:** ^1^Department of Neurology, University of Colorado Anschutz Medical Campus, Aurora, CO, USA; ^2^Department of Psychiatry, University of Colorado Anschutz Medical Campus, Aurora, CO, USA; ^3^Department of Psychology, Colorado State University, Fort Collins, CO, USA

## Abstract

Children with ASD often exhibit early difficulties with action imitation, possibly due to low-level sensory or motor impairments. Impaired cortical rhythms have been demonstrated in adults with ASD during motor imitation. While those oscillations reflect an age-dependent process, they have not been fully investigated in youth with ASD. We collected magnetoencephalography data to examine patterns of oscillatory activity in the mu (8-13 Hz) and beta frequency (15-30 Hz) range in 14 adolescents with and 14 adolescents without ASD during a fine motor imitation task. Typically developing adolescents exhibited adult-like patterns of motor signals, e.g., event-related beta and mu desynchronization (ERD) before and during the movement and a postmovement beta rebound (PMBR) after the movement. In contrast, those with ASD exhibited stronger beta and mu-ERD and reduced PMBR. Behavioral performance was similar between groups despite differences in motor cortical oscillations. Finally, we observed age-related increases in PBMR and beta-ERD in the typically developing children, but this correlation was not present in the autism group. These results suggest reduced inhibitory drive in cortical rhythms in youth with autism during intact motor imitation. Furthermore, impairments in motor brain signals in autism may not be due to delayed brain development. In the context of the excitation-inhibition imbalance perspectives of autism, we offer new insights into altered organization of neurophysiological networks.

## 1. Introduction

Autism Spectrum Disorder (ASD) is a complex disorder of brain development characterized, in varying degrees, by difficulties in social interaction, verbal and nonverbal communication, and repetitive behaviors [1]. As early as 20 months of age, children with autism exhibit a robust deficit in imitating the actions of other people [2, 3]. Diverse explanations for imitative difficulties in ASD have been proposed, including motor control [4] and sensory perception deficits [5]. Studies have found impairments in several aspects of motor function, including coordination [6], gait [7], motor imitation [8], and movement preparation [9] in both adults and children with autism. The term developmental dyspraxia has been used to describe those deficits and has been proposed to be specific to autism [10]. While delayed or aberrant fine and gross motor movements in autism used to be popularly mistaken for clumsiness, an increasing number of studies have been investigating not only the degree of impairment but also its underlying mechanism(s). Behavioral studies have investigated potential links between degree of motor impairment and types of movements and/or movement contexts in autism (for reviews, see [11, 12]. However, there is a general lack of knowledge related to deficits among neural mechanisms responsible for orchestrating movements in ASD.

Voluntary movements are accompanied by changes in cortical rhythms that can be detected by electroencephalography (EEG) and magnetoencephalography (MEG). Distinct oscillatory signals are associated with motor tasks but are differently modulated during movement imitation or observation.

First, movement-related changes in rhythmic activity in the mu-range (8-13 Hz) have been reported as early as infancy [13]. Its pre- and perimovement suppression are known as event-related desynchronization (ERD) during activation of sensorimotor areas, followed by an increase after movement onset, which has widely been reported as event-related synchronization (ERS) [14].

Second, rhythmic modulation in the ongoing beta (15-30 Hz) rhythm follows a pattern similar to the mu rhythm [15] although ERS has been more specifically named postmovement beta rebound (PMBR; [16]). It is known that many experimental factors can affect sensorimotor beta rhythms, including difficulty of the movement sequence, movement duration, and directional uncertainty (e.g., see [17, 18]). Beta oscillations may also indicate the integrity of circuit-level and neurotransmitter function. The power of PMBR has previously been associated with inhibitory brain function. For example, Gaetz et al. found that PMBR, but not beta-ERD, was correlated with the concentration of GABA measured from magnetic resonance spectroscopy in the sensorimotor cortex [19]. Others have found that using direct pharmacological manipulation of GABA-A receptors, while not having direct effects on ERD or PMBR, results in a general increase in spontaneous beta, which in turn predicts ERD and PMBR [20]. Oscillatory patterns in the beta-range of sensorimotor areas may therefore provide cortical signatures relevant to circuit dysfunction.

Third, a high-gamma band (~70-90 Hz) ERS is sometimes observed at the onset of movement [21].

Abnormalities in mu and beta rhythms have been described in ASD patients while performing motor imitation tasks, such as reduced mu-suppression during movement observation [22–24], although the relatively small sample sizes in these studies (less than 20 people per group) calls for caution in the generalization of those findings and the need for replicative studies. These observations, among others, were interpreted as supportive of a “broken mirror” theory of autism involving mirror neuron circuitry [25]. Other studies, however, have revealed no group differences in mu-band activity during action observation or imitation [26, 27]. Reduction of PMBR during action observation has been shown in adults with ASD compared to controls [28], although not in adults with Asperger syndrome [29]. To date, however, beta rhythms in children and adolescents with ASD have not been investigated during motor imitation. Oberman's group has recently extended their work on mu-suppression, reporting that it increases during childhood and adolescence and independently of an autism diagnosis [30]. Thus, developmental delay of those motor-generated oscillations, rather than deviance from typical development, does not support the “broken mirror” hypothesis in autism [25]. The strong developmental gradient in mu-suppression, as well as beta-ERD and PMBR, makes it important to distinguish studies involving children from those with adults. Although transcranial magnetic stimulation studies suggest that corticospinal motor pathways are fully developed in early adolescence [31, 32], there is other evidence suggesting further development of the motor cortex and its associated cortical oscillations well into the adolescent period. PMBR, for example, appears to strongly develop throughout adolescence. An MEG study observed limited PMBR in 4- to 6-year-olds and higher levels in adolescents aged 11 to 13, but still significantly lower compared with young adults [33]. It is also known that fine motor control in healthy children improves from birth well into early adolescence [34, 35]. It is unclear how maturational changes of the motor cortex may be affected in autism spectrum disorders. Finally, while mu and beta rhythms are generated around the same time relative to the movement but not from the same areas [36], their functional meanings are very distinct, which usually prevents from drawing conclusions based on results combined from both oscillations.

In this study, we examined mu- and beta-band oscillations in adolescents with ASD during a finger imitation task. The paradigm we chose involved simple finger-lifting imitative movement performed from the 3^rd^-person perspective and from computer-generated human hand videos. We predicted that motor-beta rhythms would be impaired in the autism group due to their motor and/or imitation problems. Specifically, we hypothesized that beta-ERD signal would be higher (i.e., greater beta suppression) in ASD because of its relevance to difficulty with movement preparation. Similarly, we expected a weaker beta-PMBR due to its association with cortical inhibitory processes, which are predicted to be impaired in autism. We expected to see lower mu-suppression in the autism group, as previously shown in the literature.

## 2. Material and Methods

### 2.1. Study Subjects

Participants were 28 right-handed adolescents (Table 1). Subjects were matched for age and intelligence quotient (FSIQ), using the 4-subtest version of the Wechsler Abbreviated Scale of Intelligence (WASI; [37]). Handedness was assessed in all subjects using the Annett Handedness Questionnaire [38]. In the ASD group, adolescents met DSM-IV criteria for ASD, as determined by consensus of the Autism Diagnostic Observation Schedule (ADOS, [39]), DSM-IV diagnosis and a parent report of ASD symptoms using the Social Responsiveness Scale (SRS; [40]) and review of all available data by a clinical psychologist (S.H.). Interobserver reliability of ADOS scores is assessed in 20% of cases, with ICCs ranging from .72 to .94. A second diagnostician independently completed a record review of 50% of cases concurred with ASD diagnosis for all cases reviewed. All subjects signed informed consent and assent to participate in the study consistent with the guidelines of the Colorado Multiple Institution Review Board.

### 2.2. Stimuli and Experimental Design

**Figure 1:**
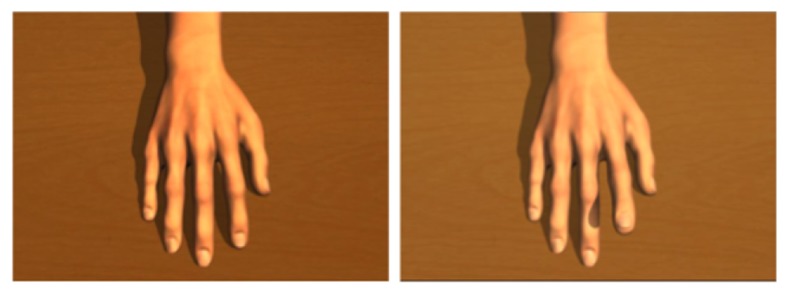
Right-hand third-person representation showing the hand at rest (left) and while performing an index lift movement (right).

The stimuli consisted of a photorealistic animated right hand, presented in the third-person perspective (Figure 1). The index or pinky fingers from this hand were lifted briefly 3 s after the beginning of the video (1 s duration for entire movement, returning to rest) every 6 s. Subjects were asked to imitate the finger movement with their right index or pinky finger as seen on the screen. Index and pinky imitation stimuli were presented in randomized order to the subject using E-prime 2.0 (Psychology Software Tools, Inc.). A total of 80 6-s trials were presented for each condition (160 total trials, for 16 minutes' total experiment duration).

### 2.3. MEG Data Acquisition, Preprocessing, and Coregistration with Structural MRI

MEG data were obtained in a magnetically shielded room (ETS-Lindgren, Cedar Park, TX, USA) using a Magnes 3600 WH whole-head MEG device (4D Neuroimaging, San Diego, CA, USA), comprised of 248 first-order axial-gradiometer sensors (5 cm baseline) in a helmet-shaped array. Five head position indicator coils attached to the subject's scalp were used to determine the head position with respect to the sensor array. The locations of the coils with respect to three anatomical landmarks (nasion and preauricular points, with the intersection of the tragus and daith of the ear defining the preauriculars) and 2 extra nonfiducial points as well as the scalp surface (approximately 500 points) were determined with a 3D digitizer (Polhemus, Colchester, VT, USA). The MEG signals were acquired continuously in a 0.1-200 Hz bandwidth and sampled at 678.17 Hz and 24-bit vertical resolution.

Single axis monolithic integrated circuits Leadless Chip Carrier (LCC) accelerometers (model ADXL103; Analog Devices, Inc.) were attached to both index and pinky fingertips in order to precisely quantify movement onset. The chips are wired to approximately 3.3 m of light weight, highly flexible, miniature cable (Cooner Wire NMVF 4/30-4046) with local bypass capacitors (0.1 uf) and encapsulated in heat-shrink. Accelerometer signals were high-pass filtered at 20 Hz, rectified, and then low-pass filtered at 10 Hz in a procedure adapted from the preprocessing of electromyography data for trigger definition [42]. The definition of movement onset was then defined as the point at which the accelerometer signals exceeded 2.5 standard deviations of the mean signal with a minimum duration between onsets of 5 s. MEG trials were defined with an epoch duration of 5500 ms, with 0 ms being the accelerometer-defined movement onset. Epochs were baseline corrected (-2500 to -1500 ms premovement onset) and those trials contaminated by excessively large MEG amplitudes (±2,000 fT) were rejected from further analysis. A mean of 96 (±29) and 106 (±17) artifact-free epochs for the autism and control groups, respectively, was used in further analyses. No group difference was observed between groups for the remaining artifact-free trials, t(26)=0.33, p=0.74. Data from excessive noise or movement artifacts were not included but small in-scanner head movements have not been corrected.

Each participant's MEG data were coregistered with structural T1-weighted magnetic resonance imaging (MRI) data prior to source space analyses (see below MRI acquisition procedures) using common landmarks from the MEG digitization procedure and MRI scan data. Structural MRI data were aligned parallel to the anterior and posterior commissures and transformed into the Talairach coordinate system [43] using the Brain Electrical Source Analysis (BESA) MRI software (BESA MRI version 2.0).

### 2.4. MEG Time-Frequency Transformation

**Figure 2:**
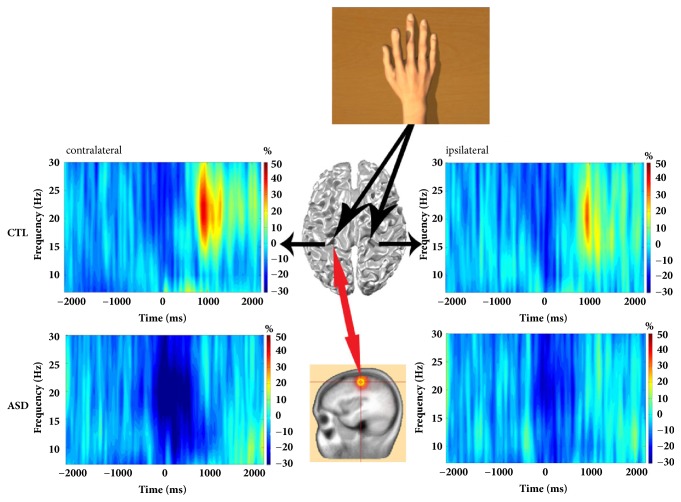
**Grand average of time-frequency spectra** of CTL (control, top) and ASD (bottom) children. TFR plots are derived from beamforming source images localized in the contralateral and ipsilateral motor areas and time-locked to movement termination (accelerometer onset). Cortical oscillations in the mu- (8-13Hz) and beta- (15-30Hz) bands showing event-related desynchronization (ERD, blue) at movement onset and postmovement beta rebound (PMBR, yellow-red) time regions are characterized. Source power during the baseline period was subtracted from source power during movement or after the movement intervals. Control subjects had a significant power decrease (ERD) in percent change from baseline in the beta frequency range beginning before movement and lasting throughout the duration of movement as well as a mu-ERD around the movement, which were followed by a strong beta power increase (PMBR) in percent change from baseline beginning shortly after movement termination. On the contrary, ASD subjects had a significantly greater power decrease (ERD) compared to controls in the beta and mu frequencies range beginning before movement and lasting beyond the movement, which was followed by a weak or no beta power increase (PMBR) beginning shortly after movement termination.

MEG postprocessing was performed using BESA 5.3 (MEGIS Software GmbH, Grafelfing, Germany). Artifact-free epochs (mean per condition: 51 +/- 12) in the time-domain were transformed to the time-frequency domain with a 2 Hz/25 ms sampling in BESA using complex demodulation [44]. This complex demodulation consists of a multiplication of the time-domain signal with a complex periodic exponential function, with a frequency equal to the frequency under analysis, and a subsequent low-pass filter. This low-pass filter is a finite impulse response filter of Gaussian shape in the time-domain, which is related to the envelope of the moving window in wavelet analysis. For our setting of a 2 Hz/25 ms time-frequency sampling, this filter has a width in the frequency domain of 5.7 Hz and in the time-domain of 79 ms full width at half maximum [45]. Time-frequency analyses were computed for each sensor individually, per trial, averaged over trials, and then normalized by dividing the power of each time-frequency bin by the respective frequency's mean baseline power. This normalization procedure allowed task-related mu (8-13 Hz) and beta (15-30 Hz) power fluctuations to be readily visualized in sensor space as the following: a decrease (blue, Figure 2) was observed priorly and extending to the movement and imaged using a -500 to 500 ms (1000 ms for mu-ERD) time window (with time 0 being the onset of the movement). Following the movement, increased beta-band power (red, Figure 2) was imaged using a 750 to 2000 ms time window. Time-frequency bins of interest were chosen to focus on maximum ERD/PMBR responses in the ipsilateral and contralateral sides of the MEG sensor array, as previously described [46, 47]. Baseline mu and beta power were extracted within a -2500 to -1500 ms time window.

### 2.5. MEG Source Reconstruction and Statistical Analyses

Using BESA Research 5.3, cortical networks were imaged through an extension of the linearly constrained minimum variance vector beamformer [48–50], which employs spatial filters in the frequency domain to calculate source power for the entire brain volume. The single images are derived from the cross-spectral densities of all combinations of MEG sensors averaged over the time-frequency range of interest, and the solution of the forward problem for each location on a grid specified by input voxel space (7 mm cubic voxels in Talairach space). Following convention, the source power in these images was normalized per participant using a prestimulus noise period of equal duration and bandwidth [48]. In the dipole-fit model used in BESA, a set of consecutive time points is considered in which dipoles are assumed to have fixed position and fixed or varying orientation. In the final analysis, we used a regional source with fixed location and orientation throughout the analysis window because this way the resulting source waveform represents the time course of activity in the region of interest. The regional source was placed on one of the first maxima of source activation, which typically was over in the motor area (identifiable by its “omega” or “epsilon” shape, as widely reported [51]). A regional source is a set of three orthogonal dipoles that represent the electrical activity of a small brain volume independent of changes of the net orientation of the equivalent dipole over time and is a robust estimator for source location, because it has only 3 degrees of freedom for the fitting procedure. To obtain the orientation of the equivalent dipole, the regional source was rotated in such a way that one of the three dipoles represented the most dipole orientation at the time of the motor-evoked peak. Then, normalized source power in the mu- and beta-range was computed from the corresponding source waveform in each ROI (left and right motor regions) against noise estimated during baseline.

Mean power was extracted between -500 and 500 ms for beta-ERD and 1000 ms for mu-ERD and between 750 and 2000 ms for PMBR. For statistical analyses, time-frequency results were subjected to group statistical analysis in a 2 x 2 x 2 mixed design ANOVA statistical test (group by finger by hemisphere) with finger and hemisphere treated as within-subjects measures. Separate ANOVAs were computed for the ERD and PMBR windows.

### 2.6. MR Data Acquisition

MR images and spectra were acquired using a 3.0T GE Signa HDx whole body, long bore MR scanner (GE Healthcare, Waukesha, WI, USA) at the Brain Imaging Center, University of Colorado Denver. Subjects were imaged in the supine position using a GE eight-channel phased array head coil. To comply with age- and population-related behaviors such as boredom and restlessness, subjects watched a movie during the exams using MR-compatible goggles and headphones (Resonance Technology Inc., Northridge, CA, USA) during the procedure. A T1-weighted sequence was acquired for tissue segmentation using a 3D inversion recovery fast, spoiled gradient echo (IR-SPGR) technique (matrix 256 x 256, FOV 22 cm, TR/TE/TI= 10/3/450 ms, NEX=1), resulting in 168 1.2 mm thick axial slices with an in-plane resolution of .86 mm^2^.

### 2.7. Finger Movement Accuracy

Behavioral analyses of correct responses were determined from the accelerometer data. Correct trials were defined as subject movements occurring on the correctly indicated finger within 3 s after the movement onset from the video displayed on the screen (i.e., from 1 s to 4 s after stimulus presentation). Subjects in both groups failed to respond with either finger on some trials, and these trials were excluded from the accuracy calculation. We analyzed this factor separately as level of responsiveness, defined as the number of trials responded to as a percentage of the trials presented. Accuracy, response times, and level of responsiveness were extracted for each participant and averaged across trials. Separate 2 x 2 ANOVA designs (group by finger, with finger as a repeated measure) were used to assess each behavioral variable.

## 3. Results

### 3.1. Behavioral Results

Participants with ASD performed their movements around 3.23 ±.24 s after the video movement onset, averaged across both fingers, while control children imitated the finger-lifting movements after 3.13 ±.12 s. No significant main effect of group was observed, *F*(1,26) = .72,* p* > .05. The group by finger interaction term was also nonsignificant, *F*(1,26) = .11,* p* > .05. This suggests the appropriateness of fixed time bins for motor-related oscillations in this specific study, since it is unlikely that group differences in motor-related oscillations were due to delayed movements in the autism group. For accuracy, the main effect of group was at the edge of statistical significance, *F*(1,26) = 1.82,* p* = .05 (Table 2). The group by finger interaction term was nonsignificant, *F*(1,26) = 1.53,* p* > .05. Finally, we looked at level of responsiveness and found that control children responded on a significantly higher percentage of trials (73.39 +/- 17.04) than their affected peers (66.48 +/- 14.91), *F*(1,26) = 6.99,* p* < .05. For responsiveness, no significant differences were noted for the main effect of finger, *F*(1,26) = 2.12,* p *> .05, or for the group by finger interaction term, *F*(1,26) = 0.69,* p* > .05.

### 3.2. Time-Frequency Results

**Figure 3:**
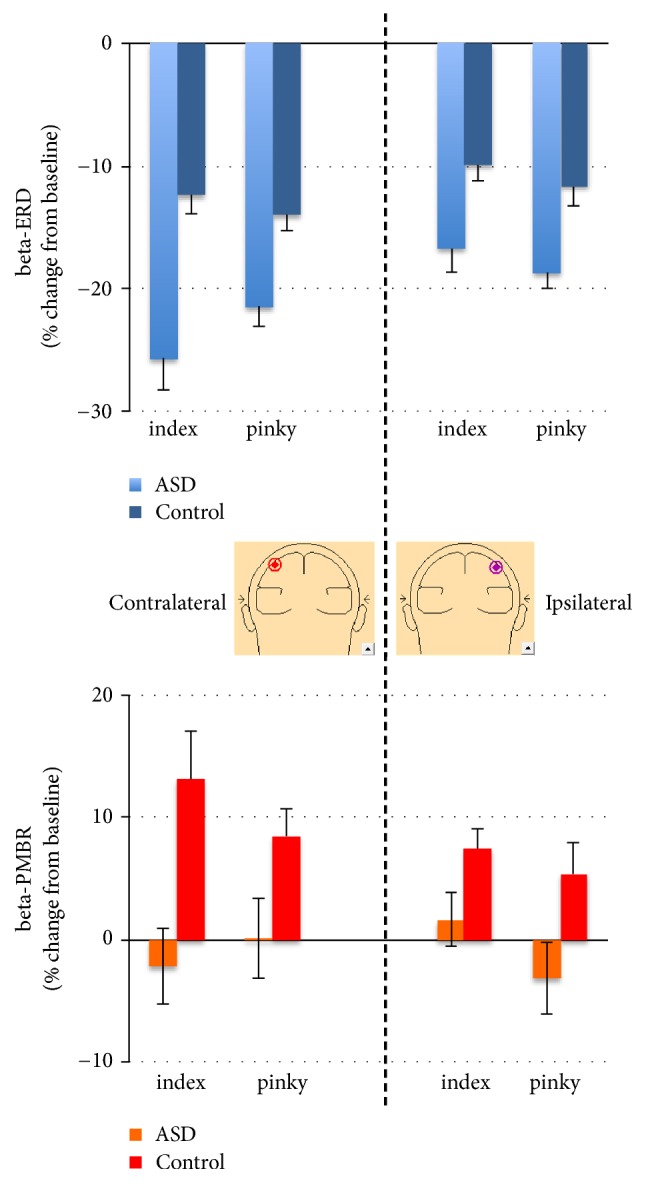
**Children with ASD exhibit greater beta-ERD than their control peers but beta-PMBR is absent.** Mean (+/- sem) beta-band ERD (top) and -PMBR (bottom) to Index and Pinky Imitation from virtual electrodes contralateral (left) and ipsilateral (right) to right hand of participant.

As expected, we found relevant motor-associated beta and mu oscillations in both hemispheres, contralateral and ispilateral to the movement, during imitation of both fingers (Figures 2 and 3).

For beta-ERD, the main effect of group was significant, *F*(1,26) = 53.02,* p* < .001, indicating greater ERD in the autism group relative to controls (i.e., greater suppression, see Figures 2 and 3, top). The hemisphere main effect was also significant, *F*(1,26) = 12.18,* p* < .001, indicating a stronger beta-ERD in the left hemisphere, contralateral to the movement. The finger main effect was nonsignificant, *F*(1,26) = .05,* p* > .05. The group by hemisphere effect was nonsignificant, *F*(1,26) = 2.14,* p* > .05. The group by finger interaction was nonsignificant, *F*(1,26) = 1.32,* p* > .05. The hemisphere by finger effect was nonsignificant, *F*(1,26) = 1.77,* p* > .05. Finally, the group by finger by hemisphere interaction was also nonsignificant, *F*(1,26) = 1.61,* p* > .05. Cohen's effect sizes were 1.1 and higher for each beta-ERD group comparison, indicating that our significant results were likely meaningful.

For PMBR, the main effect of group was significant, *F*(1,26) = 26.51,* p* < .001, indicating greater PMBR in the control group relative to the autism group (i.e., greater synchronization, see Figures 2 and 3, bottom). In contrary to the beta-ERD, the hemisphere main effect was not significant, *F*(1,26) = 2.04,* p* > .05. The finger main effect was nonsignificant, *F*(1,26) = .54,* p* > .05. The group by hemisphere effect was also nonsignificant, *F*(1,26) = .56,* p* > .05. The group by finger interaction was nonsignificant, *F*(1,26) = .86,* p* > .05. The hemisphere by finger effect was nonsignificant, *F*(1,26) = .02,* p* > .05. Finally, the group by finger by hemisphere interaction was also nonsignificant, *F*(1,26) = .62,* p* > .05. Cohen's effect sizes were 0.8 and higher for each PMBR group comparison, indicating that our significant results were likely meaningful.

**Figure 4:**
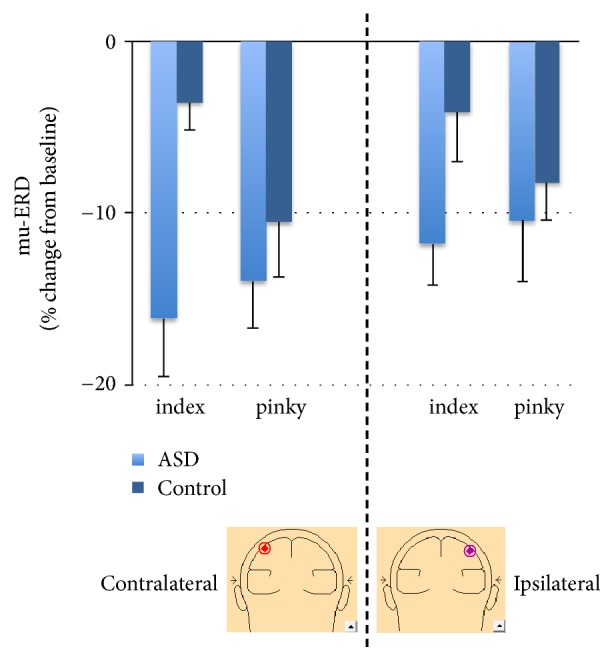
**Children with ASD exhibit greater mu-ERD than their control peers.** Mean (+/- sem) mu rhythm ERD to Index and Pinky Imitation from virtual electrodes contralateral (left) and ipsilateral (right) to right hand of participant.

For mu-ERD, the main effect of group was significant, *F*(1,26) = 9.59,* p* < .05, indicating greater mu-ERD in the autism group relative to controls (i.e., greater mu-suppression, see Figures 2 and 4). On the contrary to beta-ERD, the hemisphere main effect for mu-ERD was not significant, *F*(1,26) = 1.48,* p* > .05. The finger main effect was also nonsignificant, *F*(1,26) = .88,* p* > .05. The group by hemisphere effect was nonsignificant, *F*(1,26) = .52,* p* > .05. The group by finger interaction was marginally significant, *F*(1,26) = 3.28,* p* = 0.073, indicating that the greater mu-ERD in the ASD group was more prominent for an index movement. The hemisphere by finger effect was nonsignificant, *F*(1,26) = .06,* p* > .05. Finally, the group by finger by hemisphere interaction was also nonsignificant, *F*(1,26) = 0.21,* p* > .05. Cohen's effect sizes were 1.1 and higher for left hemisphere mu-ERD group comparison, but as low as 0.2 in the right hemisphere. This suggests that mu-suppression results may only be meaningful in the contralateral hemisphere.

**Figure 5:**
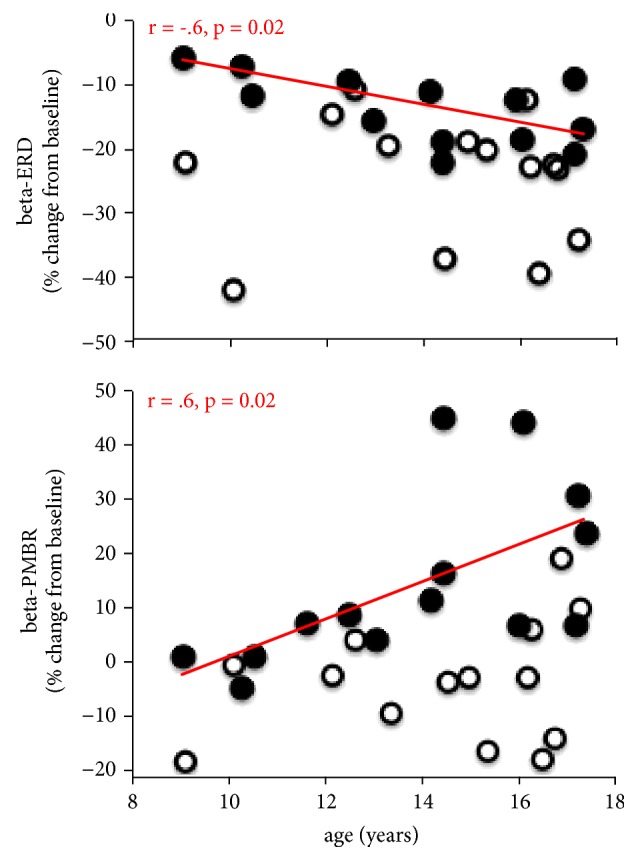
**Beta correlation results.** Representative plots of the correlation results assessing the relationship between age of the subjects and contralateral beta-ERD (top) and -PMBR (bottom) power during index imitation. The r values were -.6 for ERD and .6 for PMBR for control participants (closed black circles, red trendline). No significance was found for ASD participants (open black circles) for both ERD and PMBR.

Correlations between age and beta-ERD, beta-PMBR, and mu-ERD were examined in each group and each hemisphere using a Pearson r correlation coefficient. In the control group, there was a significant negative correlation between age and beta-ERD during imitation of the index finger,* r*(12) = -.6;* p *< .05 (Figure 5, top). It should be noted that this was only the case in the left hemisphere, contralateral to the movement. In the same group, beta-PMBR power was significantly correlated with age during index imitation,* r*(12) = .6,* p *< .05 (Figure 5, bottom), and during pinky imitation,* r*(12) = .55,* p *< .05, in the left hemisphere only. No correlation with beta power was found in the right hemisphere (*p* > .10) in the control group. In the autism group, no significant correlation was found between either beta-ERD or beta-PMBR and age for any imitated movement and any hemisphere (*p* > .10). Lastly, no significant correlation was found between mu-ERD and age for any group, any imitated movement, and any hemisphere (*p* > .10).

Baseline mu and beta power were calculated for the source reconstructed waveforms and group differences were examined using a 2-sample* t*-test. There was no significant difference between the control group and the autism group for baseline power in the beta frequency band (*p* > .10).

## 4. Discussion

In the current study, children without ASD exhibited a well-established pattern of oscillatory neural activity before and after movement onsets in brain areas associated with motor processing. Beta and mu-ERD were observed prior to movement onset and during movement execution, whereas a strong PMBR response emerged following movement termination. Those responses were observed though contralateral and ispilateral sensorimotor cortices. Children with autism also exhibited each of these neural responses, although the mu and beta power changes associated with the imitations were significantly different from those of controls. While both affected and nonaffected children were able to perform the simple action of lifting a finger, their cortical activity levels were strikingly different. In the motor cortex, induced power revealed an increase in mu- and beta-ERD and a reduction in PMBR in the ASD group compared to the control group, during imitation of both finger movements. Our results provide some physiological evidence of distinct brain activity associated with imitation of hand movements in children with autism. Below, we discuss the implications of these findings for understanding the pathological cortical activity in children with autism.

Surprisingly, we found greater mu-ERD in the group of children with autism compared to their nonaffected peers. Whether this greater ERD is restricted to the motor-related signals or rather linked to the mirror neurons remains to be clarified. Following the “broken mirror” theory of autism [25], action observation may cause the same firing effects as action execution suggesting that self-other mapping leads to imitation deficits in autism [52]. However, a recent meta-analysis provides compelling evidence that neuroimaging studies are far from providing clear support to this hypothesis [53] and neurophysiology studies on mu-suppression also show similar inconsistencies, especially with regard to two potential mu subbands [54]. Of note, mu-suppression is expected during movement observation, prior to movement execution but this was not clearly captured in our data. This could possibly be due to the method being used. While MEG studies like ours model mu source activity using an equivalent dipole, i.e., assuming that a small number of dipoles recorded mu activity [55, 56], most EEG studies reporting strong and widespread mu-suppression assume that cortical areas contributing to mu activity are distributed throughout the brain [57]. Mu data acquisition is therefore not reliably well captured using MEG instead of EEG. Alternatively, it is possible that our method prevents from collecting results from diverse sources. Beamforming uses a spatial filter designed to be fully sensitive to activity from the target voxel, while being as insensitive as possible to activity from other brain regions. Indeed, motor-related mu- and beta-ERD are generated around the same time but from distinct brain areas.

We observed significantly greater ERD in the beta-band in children with ASD. Given the beta-ERD's association with movement preparation [14] and cognitive selection of a proper motor response [58, 59], these results provide a possible physiological mechanism for the difficulty of individuals with ASD to imitate movements [60]. Increased beta-ERD has been characterized in some motor-related disorders, such as cerebral palsy [61], but a decrease has been reported in others [62, 63]. Greater beta-ERD in autism has been reported in a previous EEG study while subjects observed static hands [64], although passively watching hand actions did not produce any significant differences in beta-band activity. This difference in the static condition might suggest that, to the extent that ERD reflects movement preparation, the participants with autism had greater difficulty imagining a static hand moving, but not while actually watching the hand move. In the current study, there was no static condition and subjects did not passively watch the stimuli, so direct comparisons with the previous EEG paper are difficult. Because latency of motor responses as well as neural activity in the sensorimotor cortex during motor preparation, especially beta-band ERD, covaries with movement uncertainty [17, 65], beta-ERD may be strongly associated with movement selection. What seems clear is that beta-ERD abnormalities can be observed in autism in a variety of conditions related to movement observation or execution.

Alternatively, difficulties with body part orientation [66] or self-other mapping [67, 68] have been proposed to underlie imitation problems in autism. It is therefore possible that the third-person perspective of our imitation paradigm is partly responsible for the increased beta-ERD in the ASD group. Behavioral imitation studies have shown that, in a third-person perspective, the movement that is imitated more easily is the mirror, or specular hand, versus the anatomical hand [69]. The idea that visuospatial information processing deficits may be contributing to functional motor coordination deficits in autism has already been contemplated [70]. In this context, while beta-ERD is strongly associated with movement selection [17], a greater ERD could be due to a greater difficulty to choose which finger to move, and increased errors could have been expected in the ASD group due to a confusion of which finger is moving on the screen. We did report a weaker accuracy in the autism group, but only during the imitation of an index movement. This partly confirms the relevance of our findings to imitation problems in general in autism. Previous research has also suggested that people with autism have more difficulty when the imitated movement is meaningless or less goal-directed [71]. It is important to note that the gestures imitated in the present study were not inherently meaningful from a communication perspective. Further investigation contrasting anatomical and mirror motor imitation and exploring meaningful manipulations might provide some explanation on these aspects.

Previous studies have shown that beta-ERD power during simple finger movements is correlated with age [33, 72]. In accordance with those studies, we also obtained similar correlations although only in the control group. In children with autism, where stronger ERD is observed, no correlation was found with age. This makes sense from a developmental standpoint; cortical rhythms are resulting from synchronization of a massive number of neurons, which themselves are fully mature. Higher maturation stages in the motor cortex lead to higher beta power. But higher beta-ERD does not mean that children with ASD have a more mature brain, or both beta-ERD and -PMBR powers would be higher. In other words, the beta impairments observed in the ASD group might not likely be due to delayed maturation of the motor cortex. This confirms the dysfunctional integrative theory of autism [73]. Alternatively, the aberrant beta-ERD might be linked to the reduction in beta-PMBR. In other words, if the sensorimotor circuitry underlying beta oscillations children with autism is failing to generate synchronized beta oscillations at rest, then premovement beta-ERD would be limited by the low resting oscillatory power. However, the similar baseline beta power in both groups rules out this hypothesis in the context of our study. Similarly, while PMBR is thought to reflect an age-dependent inhibitory process [33, 72], our data showing increased PMBR are not explained by a greater age range in the ASD group.

PMBR is proposed to be associated with motor deactivation or inhibition of the motor cortex by somatosensory afferents [74], or a “resetting” of the underlying cortical networks [75]. Transcranial magnetic stimulation studies have confirmed that beta-PMBR corresponds to a period of decreased corticospinal excitability [76], suggesting that it may represent a state of cortical inhibition. It has also been suggested that several beta rhythms exist, each with a different functional significance [74, 77]. For instance, postmovement beta rebound (but not premovement beta-ERD) has been shown to be related to a prolonged period of increased corticomuscular coherence following phasic voluntary movements [78] that, in turn, may reflect the level of attention to motor performance [79]. Although a couple of autism studies report a reduction in PMBR during action observation [28, 29] but not during performance of the action, we provide evidence that postmovement beta signals are also affected during action imitation. Two explanations rise from those opposite findings. First, while we did not include an observation only condition, it is possible that neuronal circuits activated in our task proceed independently of the mirror neuron system. Second, our sample might include children with greater motor impairments than those of the other studies.

Current theories and experimental data strongly suggest that dysfunctional integrative mechanisms in ASD result from reduced neuronal synchronization [73]. The underlying cellular mechanisms seem to be an imbalance between excitation and inhibition [80], which leads to hyperexcitability and unstable cortical networks, as abnormalities in GABAergic and glutamatergic transmitter systems has been characterized in humans and animal models of autism [81, 82]. Recent studies from our group and others have demonstrated reduced GABA and increased glutamate in some regions of the brain in children with ASD [83–86] with possible evidence of reduced GABA in the motor cortex [84]. In typically developing participants, Gaetz and colleagues [19] reported a correlation between motor cortical GABA concentration and PMBR power. Consequently, it is expected that changes in cortical oscillatory rhythms, especially a reduced PMBR, will be found in the brains of ASD children. In this context, our results are consistent with the interpretation of PMBR as a marker of inhibitory neuronal signaling and the excitation-inhibition (E-I) imbalance theory of autism [80].

Finally, it is critical to consider the mixed picture of behavioral results in the current study. We found that the participants with ASD responded to the imitative stimuli less often than controls but that when the participants with ASD responded, their responses were as accurate as those in the control group. Reduced responding could be interpreted as evidence of confusion over the imitative action requested but equally could be considered evidence of greater lapsing of attention during the task. In the current study, we cannot discern between these possibilities. Since we were focused on response-locked beta-band ERD and PMBR, we could not analyze trials on which the subjects did not respond to the stimuli, limiting our understanding of whether such trials were associated with additional differences in beta-band activity.

While beta-ERD and -PBMR are generated from the same regions, it is not clear whether they result from similar events at the neuronal or network level. Our cohort of children with ASD did not exhibit significant motor defects. We interpret the aberrant pattern of beta rhythms observed in our ASD group, especially the increased ERD, as most likely associated with the difficulty of cognitive processes involved in selecting the motor response rather than with a motor deficit itself. In contrast, the reduced PMBR may be related to reduced inhibition in the motor cortices. Indeed, PMBR is absent in young children [33], which suggests that the reduced influence of inhibition in the motor cortices may represent an optimal physiological environment to facilitate motor learning or to recover from motor delay. It could therefore also be interpreted to be a compensatory consequence of the ERD changes in the autism group. Another possible interpretation of reduced PMBR in the ASD group is that of developmental delay. The mixed pattern of aberrant beta oscillations poses an interesting question for further exploration—i.e., to what extent changes in motor-beta rhythms are directly related to observable changes in motor behavior.

## 5. Limitations

Given the cross-sectional nature of the study and small sample size, we would like to warn about the highly preliminary nature of these findings. In addition, the low number of females might limit the generalization of the results. By essence, autism is a spectrum so the characteristics of children with ASD and their life circumstances are mostly heterogeneous in nature. Addressing these issues may require larger sample sizes and possibly interdisciplinary collaboration.

## 6. Conclusion

We have demonstrated that children and adolescents with autism may have reduced inhibitory drive in cortical rhythms as measured with MEG during motor imitation. Our results support previous theories that inhibitory dysfunction could be one of the factors underlying abnormal behaviors in autism. Further, changes in ERD suggest greater difficulty in movement planning in the autism group. Understanding these mechanisms may provide a potential target for future therapies to address motor-related symptoms, by both pharmacological and behavioral interventions. Whereas the relevance of altered brain oscillations to motor imitation problems in autism needs further clarification, monitoring pathological beta-bands features with MEG might hold promise as a biomarker for motor impairments in ASD. On this last point, although a large number of individuals with ASD have motor difficulties, they are not universally observed [87]. Due to this heterogeneity, specification of motor impairments in autism may be useful for the identification of clinically relevant subgroups in ASD. Moreover, a better understanding of the neurobiology of motor and/or imitation impairments is vital for identifying treatments to improve outcomes related to motor deficiencies.

## Figures and Tables

**Table 1:** Participants' characteristics.

	**ASD**	**Controls**	**χ** ^2^ ** / *t *value**	***P* value**
**N and DSM-IV diagnosis**	7 Autistic Disorder 5 Asperger's 2 PDD-NOS	14		

**Age**	14.5 ±2. 8	13.8 ±2. 8	0.66	0.52

**Male/female**	13/1	11/3	n/a	0.16

**Handedness** **∗**	0.8 ±0.3	0.8 ±0.2	0.46	0.65

**IQ**	106.5 ±19.2	110.3 ±15.8	0.57	0.58

**SRS** **∗**	104.6±21.5	n/a	n/a	n/a

**SES** **∗**	48.2±10.5	49.2±9.2	0.23	0.82

*∗* handedness scores were obtained using the Annett handedness questionnaire [38]; SRS (Social Responsiveness Scale) is a brief informant-based measurement of autism traits [40]; SES (Socioeconomic Status) scores based on the Barratt modified measure of social status [41].

**Table 2:** Behavioral results.

**Finger**	**ASD**	**Controls**
**Index**		
Reaction time ± SD	3.23 ± 0.24	3.13 ± 0.11
Accuracy ± SD	94.10% ± 6.95	98.80% ± 1.84
Responsiveness ± SD	60.70% ± 14.40	72.3% ± 13.48
**Pinky**		
Reaction time ± SD	3.24% ± 0.25	3.14% ± 0.14
Accuracy ± SD	94.00% ± 7.15	95.90% ± 6.51
Responsiveness ± SD	65.90% ± 18.16	74.80% ± 15.19

SD, standard deviation.

## Data Availability

Deidentified data is available on our laboratory server in.mat and.xlsx file format readily usable by any requester.

## Abbreviations

ASD: Autism spectrum disorders
MEG: Magnetoencephalography
ERD: Event-related desynchronization
PMBR: Postmovement beta rebound
EEG: Electroencephalography
GABA: Gamma-aminobutyric acid
DSM: Diagnostic and statistical manual of mental disorders
ADOS: Autism diagnostic observation schedule
MR(I): Magnetic resonance (imaging).

## Additional Points

*Highlights*. (i) Motor oscillations are impaired in children with ASD during motor imitation. (ii) Increased beta-ERD may be related to difficulties with movement selection and preparation. (iii) Weaker or absent beta-ERS suggests reduced inhibitory drive.
